# From theory to practice: A case study on estimating the costs of improving animal welfare on Simmental alpine dairy farms

**DOI:** 10.1371/journal.pone.0343380

**Published:** 2026-07-31

**Authors:** Agung Triatmojo, Zazie von Davier, Greta Fichter, Matthias Gauly, Thomas Zanon

**Affiliations:** 1 Faculty of Agricultural, Environmental and Food Sciences, Free University of Bozen-Bolzano, Piazza Università 5, Bolzano, Italy; 2 Rector of the University of Veterinary Medicine, Vienna, Austria; 3 Institute of Farm Economics, Thünen Institute, Braunschweig, Germany; 4 Department of Livestock Socio-economics, Faculty of Animal Science, Universitas Gadjah Mada, Yogyakarta, Indonesia; Eskisehir Osmangazi University: Eskisehir Osmangazi Universitesi, TÜRKIYE

## Abstract

This case study estimates the economic implications of enhancing animal welfare (AW) in Alpine dairy farming, specifically focusing on Simmental cattle in both conventional (CON) and organic (ORG) farms. Given the increasing demand for higher welfare standards from consumers and policymakers, it is important to assess the economic feasibility of implementing these practices. The primary aim of this study is to develop a proof of concept for estimating the costs involved in meeting specific AW standards. Data were collected from 30 dairy farms (15 CON and 15 ORG), and AW scores were evaluated using both resource-based and animal-based indicators (total score of 90 points). Additionally, AW costs were assessed by calculating the cumulative additional costs, offset by potential benefits. Results show ORG farms achieve higher AW scores (73.99) compared to CON farms (70.28) but incur significantly higher production costs (€1.263/kg ECM for ORG vs. €0.977/kg ECM for CON), primarily due to the dilution effect caused by lower milk production on ORG farms. A nonlinear relationship between AW scores and AW costs was also observed, indicating that higher AW scores in initial farm conditions could, in some cases, lead to cost savings, particularly in larger and more efficient farms. The study emphasizes the need for clearly defined AW standards and a corresponding scoring system that reflects both the welfare impacts on animals and the economic feasibility for farmers. Further, we argue that the aggregation of AW scores should not merely categorize welfare on a simple best-to-worst continuum. Rather, it should account for the specific targets and conditions that policymakers and consumers are willing to support financially.

## Introduction

There has been growing awareness among consumers and policymakers about the need to improve animal welfare (AW) standards in farming [1]. However, economic concerns continue to pose significant challenges for farmers, as the costs of implementing AW measures often outweigh the perceived benefits, particularly when these costs are not offset by market rewards or policy incentives [2]. In Germany, for instance, farmers are offered incentives by retail chains for meeting specific AW standards [2,3], but there is no clear calculation showing that these incentives adequately cover the costs involved. The challenge of estimating the costs associated with improving AW has been widely debated. Some scholars argue that AW cannot be defined solely by human interests [4], while the intangible impacts on animals are often difficult to measure due to data limitations [5]. Monetizing AW is essential for avoiding arbitrary decision-making, as it provides a common scale for evaluating the outcomes of AW improvements [4]. Money serves as a convenient unit of measurement because the costs incurred by producers to implement AW standards are easily quantifiable. Given its central role in policy debates, expressing the impacts of AW policies in monetary terms allows for direct comparison and better-informed decision-making [6].

Monetizing AW can be approached from two main perspectives: anthropocentric and non-anthropocentric. The anthropocentric approach monetizes the impacts of AW improvements insofar as they affect producers and consumers [5], often using methods like Cost-Benefit Analysis (CBA) [7–10] and Willingness to Pay (WTP) [11–13]. In contrast, the non-anthropocentric approach prioritizes the value of impacts on animals themselves, assessing the extent of harm relative to normal welfare conditions. Non-anthropocentric monetization often uses Social Welfare Functions (SWF), which adapt the concept of quality-adjusted life years (QALYs) for animals [4]. However, the application of SWFs in practical policy evaluations remains limited, and this approach is not currently included in the EU’s policymaking guidelines [5,14]. Nonetheless, using CBA to monetize AW is seen as a promising long-term solution, as it quantifies the intangible impacts of AW improvements in dairy farming, such as those related to lameness [15], longevity [16], and health [17].

Several AW protocols (structured guidelines for assessing and ensuring animal well-being through indicators and interventions) have been developed to integrate both human and animal perspectives [18]. These protocols may use qualitative nominal or ordinal scales to classify indicators into categories (e.g., presence/absence or insufficient/acceptable/excellent), while quantitative ratio scales assign numerical values to measurable factors for more precise assessment. For example, the Welfare Quality® Protocol is an ordinal-scale framework in Europe, while Italy’s ClassyFarm system also evaluates ordinal-scale animal welfare in dairy farming, focusing on biosecurity, farm management, animal-based measures, and hazard alarm systems [19,20]. However, these protocols partly lack clear thresholds for interpreting welfare scores, particularly in smaller herds where scores may be skewed [20]. Recent advancements have moved from qualitative nominal and ordinal scales (e.g., Katzenberger et al. [21]) to quantitative ratio scales, such as the work by Poulopoulou et al. [22], which assigns proportional scores to specific AW indicators. However, these methodologies lack clarity in terms of how different indicators are weighted, creating a gap that this study aims to identify. Building on the work of Vissers et al. [23], this study aims to develop a method for monetizing AW by correlating welfare scores derived from the AW protocols of Katzenberger et al. [24] with AW costs (defined as the net expense of implementing AW measures after accounting for potential benefits) instead of milk production costs. The objective of this study is to provide a proof of concept for estimating the costs of improving AW on conventional and organic Simmental Alpine dairy farms. Estimating these costs could provide useful insights for supporting farmers’ decision-making and assisting policymakers in determining additional costs associated with AW regulations, a growing focus in both scientific research and policy discussions [6,25].

## Materials and Methods

### Study region

South Tyrol is an alpine province in Northern Italy, where grassland is the primary type of cultivation. The region has a total of 61,332 hectares mown for livestock feed and 127,216 hectares used as mountain pastures [26]. In 2024, the dairy cattle farming sector, consisting of 3,967 farms with an average of 15 cows per farm, generated a total milk production of 365.5 million kilograms, with each farm producing an average of 92,143 kilograms [27]. The most common dairy cattle breeds in the region are Simmental and Brown Swiss, each accounting for 30% of the population, while Holstein Friesian makes up approximately 20%. The study focused exclusively on farms that raised purebred Simmental cattle, as this breed is commonly used in the region and is particularly suitable for small-scale alpine dairy farming [28]. In the Alps, mixed dairy–beef or dual-purpose breeds like Simmental are still widely used due to their adaptability to the challenging production conditions of these farms [29]. Further, dual-purpose breeds can remain economically competitive in mountainous regions due to the value of calves, cull cows, and subsidies, even though they produce lower milk yields compared to milk-specialized breeds [28]. The organic farms were certified according to EU regulations on organic production [30], adhering to a hay milk production system that limits the use of concentrate feed to a maximum of 25% of the annual feed ratio, with no silage feeding (EU-Regulation 2016/304). It is important to note that the sample farms visited do not directly represent all mountain dairy farms in South Tyrol but rather reflect a range of farming systems. This diversity arises largely from the significant geographic variability found across mountain regions, which influences farming practices and conditions.

### Data collection and ethical considerations

The recruitment period for this study began on **01/05/2023** and concluded on **27/10/2023**. A team of auditors from the Free University of Bolzano, including trained veterinarians and researchers, visited 30 mountain dairy farms: 15 conventional (CON) and 15 organic (ORG), each with uniform herd size and grassland. These farms were selected in collaboration with the South Tyrolean breeders’ associations (*Vereinigung der Südtiroler Tierzuchtverbände*). The association sent an invitation and an introduction about the study to the farmers. Those who voluntarily agreed to participate confirmed their participation by phone with the association, after which the surveyor visited their farms. The initial informed consent was then witnessed by a representative of the association, and the authors did not have access to or provide these data.

The study complied with the Research Ethics Committee of the Free University of Bozen-Bolzano, adhering to the *Europäische Richtlinie* 86/609/EEC and the European Data Protection Regulation (GDPR 2016/679). Given the non-invasive, non-clinical nature and design of the study, which involved only adult participants engaged in agricultural activities, formal ethical approval was not required. The questionnaire did not record personal identifiers, ensuring participant anonymity throughout data handling and analysis. All procedures involving human participants complied with the Declaration of Helsinki, and no foreseeable physical, psychological, or social risks were identified for the participants. The animal welfare assessment involved monitoring animals from a distance to prevent any impact from the observation. At the end of each interview, verbal declaration was obtained directly from participants as confirmation of their voluntary participation.

### AW assessment

A maximum of ten randomly selected lactating dairy cows from each farm were assessed using an AW protocol developed by Katzenberger et al. [24] for mountain dairy farms. This protocol is based on the EU-funded Welfare Quality project and the guidelines of the German association *Kuratorium für Technik und Bauwesen in der Landwirtschaft* e.V. (KTBL) [31], with modifications to address the specific operational conditions of small-scale farms [32]. The protocols scale were then converted from an ordinal to a ratio using the benchmarking tool developed by Poulopoulou et al. [22] for assessing dairy cattle welfare on small-scale farms (Table 1). Each indicator was assigned a score based on specific conditions. Resource-based indicators included water supply (2 for 1 drinker/2 cows, 0 for fewer), water flow rate (>10 L/min = 4, 5–10 L/min = 2, < 5 L/min = 0), cleanliness of drinkers (4 for all clean, 2 for half dirty, 0 for all dirty), and lying space usage (≥1:1 = 10, 0.9:1 = 5, < 0.9:1 = 0). Animal-based indicators included BCS (0 for very thin, 5 for thin, 10 for normal, 0 for very fat), cleanliness (5 for udder not present, 3 for upper hind leg clean, 2 for lower hind leg clean), and skin alterations (3 for no alterations on neck, 4 for no alterations on hock). Additional indicators included avoidance distance (<1 m = 10, > 1 m = 0), claw condition (10 for no alterations, 0 for any alteration), locomotion (10 for no lameness, 0 for heavy lameness), and dystocia occurrence (10 for none, 0 for present). The individual scores were summed to generate an overall score, with higher values indicating better animal welfare. In comparison to the study by Poulopoulou et al. [22], which had a maximum score of 100, our study did not sufficiently assess the rising behavior indicators. Consequently, the maximum possible score in our study was 90 points.

**Table 1:** Description of the benchmarking system with a 10-point scale (0 = worst; 10 = best) for the resource- and animal-based indicators used to calculate the farm animal welfare index at herd level, based on the study of Poulopoulou et al. [22].

Variable	Criteria	Score
**Resource-based indicators**		
Water supply (10)		
Number of working drinkers	1 drinker/2 cows	2
	Fewer than 1 drinker/2 cows	0
Waterflow rate	>10 L/min	4
	5-10 L/min	2
	<5 L/min	0
Level of cleanliness of the drinkers	All drinkers are clean	4
	Half of the drinker are dirty	2
	All drinkers are dirty	0
Lying space usage (10)	Box ratio ≥1:1	10
	Box ratio 0.9:1	5
	Box ratio <0.9:1	0
**Animal-based indicators**		
BCS (10)	Very thin (1)	0
	Thin (2)	5
	Normal (3)	10
	Fat (4)	5
	Very fat (5)	0
Cleanliness (10)		
Udder	Not present	5
	Present	0
Upper hind leg	Not present	3
	Present	0
Lower hind leg	Not present	2
	Present	0
Skin alteration (10)		
On neck	No alterations	3
	Hairless spots	2
	Open wounds and/or swellings	0
At the carpal	No alterations	3
	Hairless spots	2
	Open wounds and/or swellings	0
At the hock	No alterations	4
	Hairless spots	2
	Open wounds and/or swellings	0
Avoidance distance (10)	Touching the muzzle	10
	<1 m	10
	>1 m	0
Status of the claws (10)		
Front claw	No alterations	10
	Any form of alteration	0
Rear claw	No alterations	10
	Any form of alteration	0
Locomotion (10)		
Lameness at rest/standing	No lameness	10
	Slight lameness	5
	Heavy lameness	0
Lameness in motion	No lameness	10
	Slight lameness	5
	Heavy lameness	0
Dystocia occurrence (10)	None present	10
	Present	0

### AW standard scenario

**Fig 1:**
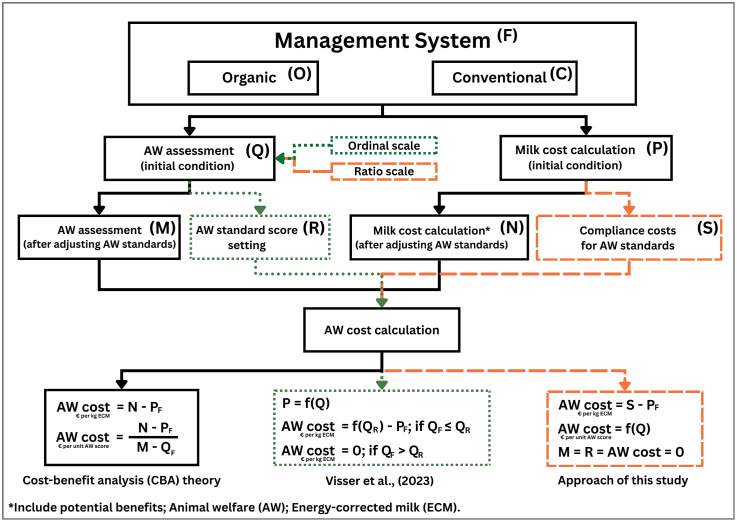
Flow diagram method of the study.

**Fig 2:**
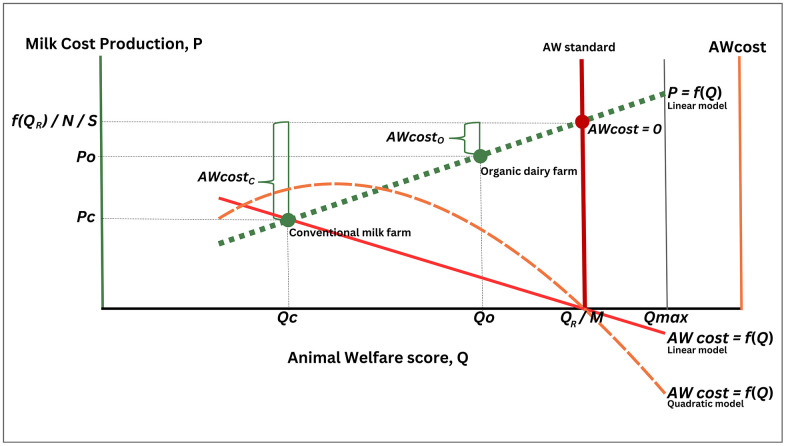
Illustration of the cost-function for AW score and milk cost of production (green-dot line) and AW cost (orange-dash line) for a conventional and organic farm. Modified from Vissers et al. [23].

Animal welfare (AW) standards are defined as the minimum criteria that ensure animals experience only limited negative impacts on their well-being. These criteria may vary based on the political consensus and community values. In this study, the AW standard score represents the threshold at which no additional cost is incurred to meet a particular standard [23]. AW standards can be categorized into different levels, such as Level 1–3, moderate to premium, or bronze to gold [3]. However, due to the challenges in defining multiple levels of standards, this study focuses on a single standard and excludes higher-level scenarios. At the time of data analysis, no official AW standards were defined in Italy. Therefore, this study adopted the AW standards or criteria from the AG Rind of the *Kompetenznetzwerk Nutztierhaltung* [33], as the AW protocol in this case study aligns with the German KTBL guidelines. The adjustments from the initial conditions to the AW standard primarily concern resource-related and management-related indicators, as these directly influence improvements in animal welfare. Given the extensive criteria for AW standards, only those not met in the initial scenario are presented in Table 2. Since the AG Rind criteria do not specify a fixed AW standard score, a threshold was not predetermined in this study. Instead, AW cost was used to estimate the threshold, defined as the point where the cost of meeting the AW standard equals zero, as illustrated in Figs 1 and 2.

**Table 2:** AG Rind criteria for AW standard scenarios in this case study.

Criterion	
**Resource-based indicators**	
Housing	• Free stall housing or tethering with the possibility of movement
Space requirements	• 6 m² covered, usable floor area per cow
	• Exception for covered stall systems with covered lying boxes/feeding places, such as cubicle systems
	• Animal-to-lying space ratio: 1:1
	• Animal-to-feeding space ratio: 1.5:1
	• Width of free passages with drinkers/brushes: at least 3.0 m
	• Aisle width at the feeding table: at least 3.0 m
	• Aisle width at the lying boxes: at least 2.5 m
	Dead-ends aisles < 3.0 m width, up to 6 lying boxes allowed
	Max. 10% of lying places in dead-end aisles
Functional areas	• Bedding mattress ≥ 5 cm
	• Walking and feeding areas with solid or perforated flooring (non-slip, safe for walking)
Selection Criteria	• Lying box width: 1.20 m
	• At least one cow brush or adequate scrubbing device per 60 animals
**Management-based indicators**	
Grazing access	• Grazing access (120 days/6 h)
On-farm Self-Assessment	• Farm self-control according to KTBL animal welfare indicators
Animal health	• Documented routine hoof care twice a year• Selective antibiotic according to regulation
Further training for animal caregivers	• Participation in training courses

Further details are available in Tergast (2023).

### Milk cost calculation

#### Cost at initial condition.

The farmer interview was conducted using a detailed questionnaire covering various aspects of farm operations, including basic agricultural production data, farm structure, farm economy, and management information. The questionnaire focused on all farm inputs and outputs. Expenditures considered include costs related to crop and feed production (including land costs), purchased feed, machine and building maintenance, and other expenses such as energy, insurance, veterinary services, artificial insemination, claw trimming, performance testing, animal transport (purchase, sale, or slaughterhouse), bedding, miscellaneous costs, and consulting fees. Depreciation accounts for costs associated with building construction or renovation and investments in machinery, with amortization periods set at 30 years for farm buildings and 15 years for agricultural machinery. Labor costs are calculated by multiplying the estimated working time for daily tasks by the standard agricultural wage in South Tyrol (€15.75 per hour), as outlined by [34]. Specifically, 164.2 hours per cow per year are estimated for tie-stall farms, and 112.6 hours per cow per year for loose housing farms [35]. Capital costs refer to the initial investment required to acquire, maintain, and improve the farm’s fixed assets, including all payments and imputed interest.

#### Compliance costs for AW standards.

Due to the cross-sectional nature of the data, it is not possible to directly observe changes in AW scores and milk production costs following adjustments to AW standards. Therefore, this study benchmarks compliance costs based on the methodology of Tergast (2023), adjusting for farm-level factors such as grazing implementation, daily task working time, and barn reconstruction (Table 2). Compliance costs refer to the total expenses incurred after meeting a specific AW standard, while AW costs refer to the cumulative additional costs or losses offset by potential benefits or savings (Fig 1). Due to differences in farm size and regional economic contexts compared to the benchmark study, a farm representing best practices in each management system was selected as the reference for estimating compliance costs in this study.

Thanks to the detailed questionnaire, farm-specific calculations were made to reflect the additional costs and potential benefits, considering the varied initial conditions. While milk price and mortality scenarios remain constant, benefits such as optimized working hours and reduced feed and veterinary costs associated with grazing were incorporated into the analysis. For dairy farms with grazing access that did not meet the criteria, a standardized additional cost based on Peratoner et al. [36] was applied to account for mountainous topography and remote, steep pastures. This study followed focus group observations from Tergast et al., [10], which indicate that the improving grazing access (120 days/6 hours per day; Table 2) initially increases milk yield at the start of pasture access, but this yield gradually declines over the grazing season, resulting in an overall reduction of approximately 1,000 kg ECM per cow per year. This pattern aligns with performance changes observed when farms transition from summer grazing to year-round housing and is consistent with comparative studies on pasture versus barn systems [35,36]. However, it is acknowledged that the extent of yield reductions may vary across farms depending on factors such as farm management practices, cow breed, pasture quality, and climate conditions [37,38]. These factors represent limitations of the case study, as the assumptions may not be universally applicable to all farm types. Additionally, barn reconstruction costs were included in the compliance cost calculation for farms with a lying space ratio below 0.9:1, based on the estimated costs required to rebuild barns to meet the specified scenario [10]. However, it should be noted that the study did not consider the benefits of cow comfort, such as improved lying space [39] and bedding [40], which could lead to increased milk production.

### Statistical analysis

The AW score and milk production cost data were analysed using general linear models (GLM) with the management system (CON, ORG) as a fixed factor and the farm as random factor. Confounding factors were included in the analysis, assuming these factors remained constant across farms. These factors include elevation, number of calves, cow lactation status, dairy cow health, permanent grassland, the opportunity cost of unpaid labor, and depreciation costs (S1 Table). The assumptions of the model were assessed to ensure its validity. First, the normal distribution of the residuals was tested using the Kolmogorov-Smirnov Test. Second, the homogeneity of variance was assessed using the Breusch-Pagan test. To explore the relationship between AW scores and AW costs, a quadratic cost function was analyzed, comparing the linear and quadratic regression models to identify the best fit (Fig 2). We hypothesize that a higher AW score correlates with lower AW costs, suggesting that better animal welfare practice at initial farm condition may incur lower additional cost. As emphasized earlier, AW standard score, or threshold, will be derived from the quadratic function at the point where the AW cost equals zero. This point indicates that no additional costs are incurred to achieve the selected AW standard. All analyses were conducted using STATA 17.

### Descriptive data of the farms

The farm characteristics presented in S1 Table are derived from the dataset first published in Sabia et al. [41], which focused on environmental efficiency and carbon sequestration potential. In particular, CON farms were located at an average elevation of 1,148 meters above sea level (m.a.s.l.) (± 189 SD), while ORG farms were situated at a slightly higher elevation, with a mean of 1,218 m.a.s.l. (± 246 SD). Both farm types had similar land areas under permanent grassland, with an average of 12.4 hectares (± 4.9 SD) for CON farms and 12.4 hectares (± 5.3 SD) for ORG farms. In terms of stocking rate, CON farms had an average of 1.53 livestock units (LU) per hectare (± 0.37 SD), while ORG farms had a slightly lower stocking rate at 1.31 LU per hectare (± 0.28 SD). Regarding herd size, CON farms typically housed 17.5 dairy cows (± 5.2 SD), while ORG farms had an average of 16.1 cows (± 7.9 SD). The number of cuts from permanent grassland was more frequent in CON farms, with an average of 3.14 cuts (± 1.1 SD) compared to 2.0 cuts (± 0.72 SD) for ORG farms. When it comes to milk yield, CON farms produced more energy-corrected milk (ECM) per cow per year, with an average of 9,505 kg (± 912 SD), while ORG farms produced 6,540 kg (± 1,336 SD) of ECM per cow per year (S1 Table).

## Results

### AW score

#### Resource-based indicators.

The comparison of resource-based indicators between CON and ORG farms revealed some differences in water supply and drinker conditions. Tie-stalls were used in 4 of the 15 CON farms and 2 of the 15 ORG farms, while the remaining farms in each group (11 CON, 12 ORG) had free-stall barn systems (S1 Table). On average, CON farms had 0.9 (± 0.7 SD) trough drinkers and 3.5 (± 3.5 SD) bowl drinkers, while ORG farms had 1.4 (± 0.9 SD) trough drinkers and 1.73 (± 1.7 SD) bowl drinkers. In terms of water supply, 11 of the 15 CON farms had sufficient water flow rates (≥ 10 L/min), and 13 farms had clean drinkers. In comparison, all ORG farms had sufficient water flow rates, and 14 farms had clean drinkers (S1 Table). When scored on the AW scoring system, water supply was rated 7.86 for CON and 9.07 for ORG, with a p-value of 0.059, indicating no significant difference at the 5% level. The number of working drinkers was 1.20 in CON and 1.33 in ORG farms (p-value = 0.704), showing no significant difference. The water flow rate was scored 3.47 for CON and 4.00 for ORG farms, with a p-value of 0.020, indicating a statistically significant higher water flow rate in ORG farms. Similarly, the level of cleanliness in the scored drinkers was 3.20 for CON and 3.73 for ORG (p-value = 0.273), showing no significant difference. Finally, lying place usage was 2.73 for CON and 1.65 for ORG, with a p-value of 0.281, indicating no significant difference (Table 3).

**Table 3:** Least square means, p-value and standard error of means (SE of means) of the AW score of the conventionally (CON) and organically (ORG) managed farms.

Indicator	Management system		p-value	SE of means
	CON	ORG		
Resource-based indicators				
Water supply (10)	7.86	9.07	0.059	0.634
Number of working drinkers (2)	1.20	1.33	0.704	0.351
Waterflow rate (4)	3.47	4.00	**0.020**	0.228
Level of cleanliness of the drinkers (4)	3.20	3.73	0.273	0.486
Lying place usage (10)	2.73	1.65	0.281	1.001
Animal-based indicators				
BCS (10)	8.00	8.67	0.434	0.852
Cleanliness (10)	6.43	6.78	0.668	0.807
Udder (5)	4.02	4.03	0.986	0.389
Upper hind leg (3)	1.53	1.81	0.333	0.282
Lower hind leg (2)	0.88	0.95	0.761	0.219
Skin alteration (10)	8.53	9.62	**0.000**	0.257
On neck (3)	2.95	3.00	0.059	0.024
At the carpal (3)	2.61	2.92	**0.000**	0.078
At the hock (4)	2.96	3.70	**0.000**	0.202
Avoidance distance (10)	10.00	9.93	0.301	0.064
Status of the claws (10)	8.04	8.67	0.399	0.742
Front claw (10)	8.11	8.81	0.371	0.783
Rear claw (10)	7.97	8.53	0.494	0.819
Locomotion (10)	8.67	9.61	**0.001**	0.288
At rest/standing (10)	8.83	9.70	**0.007**	0.321
In motion (10)	8.48	9.50	**0.001**	0.291
Dystocia occurrence (10)	10	10	–	–
Total AW score (90)	70.28	73.99	**0.046**	2.13

Indicators are derived from the AW protocol outlined by Katzenberger et al. [24] and evaluated using the scoring system developed by Poulopoulou et al. [22]. The numbers in parentheses show the maximum score for each indicator or parameter (with higher scores indicating better AW conditions), with claw status and locomotion scores presented as averages.

#### Animal-based indicators.

The comparison of animal-based indicators between CON and ORG farms revealed both significant and non-significant differences, with a higher score indicating a higher AW condition. Body Condition Score (BCS) was 8.00 for CON and 8.67 for ORG (p-value = 0.434), showing no significant difference (Table 3). Similarly, cleanliness scores for CON (6.43) and ORG (6.78) did not differ significantly (p-value = 0.668). The udder cleanliness score was 4.02 for CON and 4.03 for ORG (p-value = 0.986), showing no significant difference. Other indicators, such as cleanliness of the upper hind leg (1.53 CON vs. 1.81 ORG, p-value = 0.333), lower hind leg (0.88 CON vs. 0.95 ORG, p-value = 0.761), and avoidance distance (10.00 CON vs. 9.93 ORG, p-value = 0.301), also showed no significant differences. In contrast, skin alterations were less severe in ORG farms (9.62 vs. 8.53, p-value = 0.000), with significant differences also observed for carpal (p-value = 0.000) and hock (p-value = 0.000), where higher scores indicate improved AW. Significant differences were found in locomotion (9.61 ORG vs. 8.67 CON, p-value = 0.001), rest/standing behavior (9.70 ORG vs. 8.83 CON, p-value = 0.007), and movement (9.50 ORG vs. 8.48 CON, p-value = 0.001), all favoring ORG farms, reflecting better AW conditions (Table 3).

**Fig 3:**
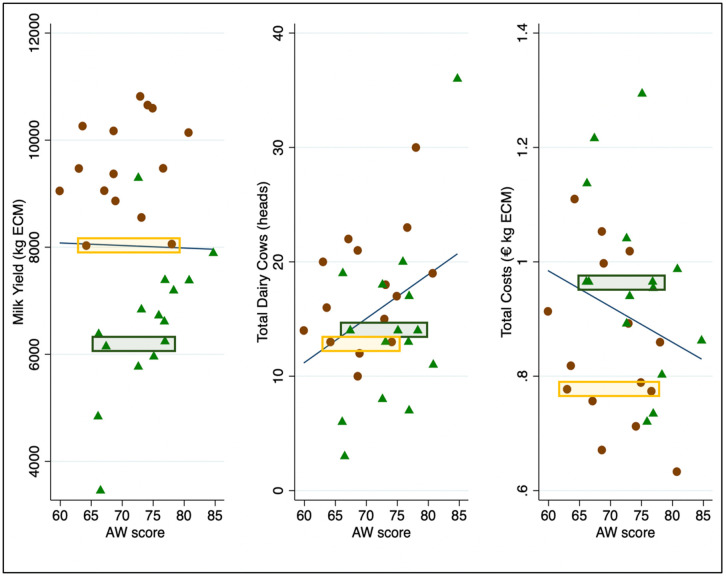
Scattered plot of total AW score per farm depending on selected farm characteristics. Data plot within the rectangle illustrates that substantial variations in AW scores occurred despite similar farm performance.

**Fig 4:**
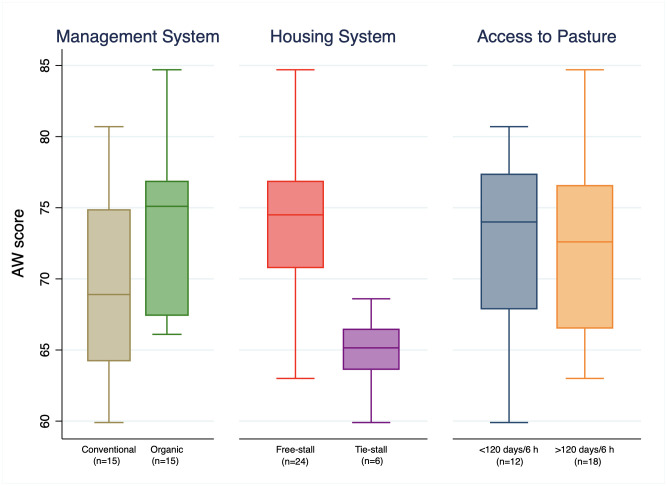
Boxplot of total AW score per farm depending on management system, housing and access to pasture.

Overall, the total AW score was significantly higher in ORG farms (73.99) compared to CON farms (70.28), with a p-value of 0.046. However, most individual indicators did not reach significance. Given the number of comparisons, this borderline significance should be interpreted with caution. Figs 3 and 4 show the variability in AW scores across farm characteristics, management systems, housing systems, and access to pasture. Even under similar performance, there were substantial variations in AW scores (Fig 3). ORG farms exhibited higher AW scores and greater variability compared to CON farms. Regarding housing systems, free-stall farms showed the greatest variability, while tie-stall farms had the least. Additionally, farms with over 120 days of pasture access exhibited more variability in AW scores compared to those with less than 120 days (Fig 4).

### Milk cost of production

In the initial condition, operating costs were lower for CON farms (€0.391/kg ECM/year) compared to ORG farms (€0.512/kg ECM/year), as shown in Table 4. Significant differences were observed in the costs of homegrown feed, purchased feed, and energy, while no significant differences were found for veterinary costs, artificial insemination, dairy-related expenses, and machinery and building upkeep. In terms of total fixed costs, ORG farms incurred significantly higher costs (€0.752/kg ECM/year) compared to CON farms (€0.586/kg ECM/year), primarily due to herd renewal purchases, insurance, and other costs. However, no significant differences were found for fees and consulting or the opportunity cost of family labor. When adjusting for AW standards, there were no significant differences in purchased feed and energy, as CON farms are expected to use more pasture and lower energy to control the environment in the barn (Table 4). However, significant differences were observed in compliance costs, with ORG farms tending to have more cost savings (indicated by negative values compared to CON farms), reflecting lower costs required to meet AW standards. Nearly all ORG farms met the requirements for pasture access, though both groups still needed to upgrade barns to comply with the defined criteria.

**Table 4:** Least square means of economic performance at initial condition, compliance cost for AW standards, and cost or cost savings of the CON and ORG groups (n = 15, respectively).

Items	Initial condition*		Compliance costs*		Costs or cost savings**	
	CON	ORG	CON	ORG	CON	ORG
Costs						
Operating costs	**0.391**	**0.512**	**0.447**	**0.512**	**5.38**	**0.35**
Home grown feed	**0.128**	**0.163**	**0.138**	**0.164**	**0.92**	**0.25**
Purchased feed (concentrate)	**0.146**	**0.206**	0.172	0.206	**2.64**	**−0.03**
Veterinary cost	0.007	0.013	0.008	0.012	**0.09**	**−0.02**
Artificial insemination	0.007	0.009	0.009	0.008	**0.06**	**−0.02**
Dairy related expenses	0.024	0.020	0.027	0.019	**0.18**	**−0.01**
Energy (fuel, electricity)	**0.046**	**0.060**	0.055	0.057	**0.91**	**−0.17**
Machinery and building upkeep	0.033	0.043	0.039	0.043	**0.71**	**0.03**
Fixed costs	**0.586**	**0.752**	**0.783**	**0.929**	**19.59**	**18.00**
Depreciation	**0.155**	**0.217**	**0.297**	**0.396**	**14.30**	**18.10**
Building	**0.082**	**0.109**	**0.221**	**0.293**	**13.79**	**18.55**
Machinery	**0.073**	**0.108**	**0.076**	**0.103**	**0.51**	**−0.45**
Herd renewal purchases	**0.055**	**0.079**	**0.059**	**0.076**	**0.63**	**0.01**
Insurance	**0.044**	**0.100**	**0.051**	**0.101**	**0.48**	**0.02**
Fees and consulting	0.011	0.016	0.014	0.016	**0.12**	**−0.01**
Other cost	**0.029**	**0.048**	0.030	0.048	**0.21**	**−0.01**
Opportunity cost of family labour	0.304	0.279	0.345	0.277	**3.89**	**−0.22**
Total costs	**0.977**	**1.263**	**1.229**	**1.442**		
Revenue						
Milk price	**0.591**	**0.787**	**0.591**	**0.787**	–	–
Cattle sales	0.080	0.091	0.094	0.096	**1.46**	**0.52**
Subsidies	**0.071**	**0.122**	**0.081**	**0.128**	**1.16**	**0.68**
Total revenue	**0.741**	**0.999**	0.767	1.010	**2.52**	**1.27**
AW costs					**20.12**	**9.61**
Returns						
Level 1 (milk price – TC)	−0.386	−0.477	−0.639	−0.657		
Level 2 (milk price+cattle sales – TC)	−0.306	−0.386	−0.544	−0.561		
Level 3 (milk price+cattle sales+subsidies – TC)	−0.236	−0.265	−0.462	−0.431		
Level 4 (milk price – OC)	**0.200**	**0.275**	**0.144**	**0.274**		
Level 5 (milk price+cattle sales+subsidies – OC)	**0.350**	**0.490**	**0.320**	**0.499**		

Bold mean value indicates significant different at 5%. *€/kg ECM/year. **€ cents/kg ECM/year.

Regarding compliance costs to meet AW standards, both groups are expected to incur higher losses compared to the initial condition, as potential benefits and additional subsidies do not fully offset the costs required for returns at Levels 1, 2, and 3 (Table 4). If we assume no increase in milk price, CON farms will experience a reduction in returns at Levels 4 and 5 due to lower milk production resulting from the shift to a grazing system. In contrast, ORG farms are expected to maintain relatively stable, slightly increased returns, benefiting from higher subsidies and stable milk production. Irrespective of compliance costs, the farm-gate milk price in South Tyrol is higher compared to other Alpine regions (Table 5), but it still results in a loss for both groups, as the production costs are higher in this case study (Table 4).

**Table 5:** Farm-gate milk price for CON and ORG farm in selected European alpine regions (€/100 kg) from 2021-2024.

Region, Country	CON				ORG			
	2021	2022	2023	2024	2021	2022	2023	2024
European Union (27)	36.74	50.10	46.95	48.28	36.81	50.22	46.99	48.42
Lombardy, Italy^1^	37.16	49.38	51.33	51.34	51.14	62.03	60.28	60.68
South Tyrol, Italy (Hay-milk)	50.17	58.15	68.68	67.63	68.32	76.33	87.00	85.80
Austria (Hay-milk)^2^	35.08	46.03	48.36	46.76	50.61	59.02	64.48	60.10
Bavaria, Germany	36.58	51.85	49.72	49.48	50.06	57.20	57.58	57.56
Rhône-Alpes, France^3^	39.90	46.18	50.17	50.10	46.12	46.71	49.15	49.30
Switzerland	63.13	73.44	76.64	77.09	76.09	87.68	93.71	96.83

* 3.7–4.0% fat content and 3.2–3.4% protein content; Value-Added Tax (VAT) not included; Source: Clal.it (2025), Agrarmarkt.ch (2025), Zentrale Milchmarkt Berichterstattung GmbH (ZMB), Bundesanstalt für Landwirtschaft und Ernährung (BLE), estimations made by Agrarmarkt Informations-Gesellschaft mbH (AMI; 2025), FranceAgriMer (2025).

^1^Italian “spot” organic milk (stored in bulk tanks, delivery to dairy Northern Italy), as determined by the Milano Monza Brianza Lodi Chamber of Commerce.

^2^Weighted averages of farm-gate regular raw conventional and organic milk prices from all regions in Austria.

^3^Weighted averages of farm-gate regular raw organic milk prices from all regions in France

#### Compliance costs for AW standards.

**Fig 5:**
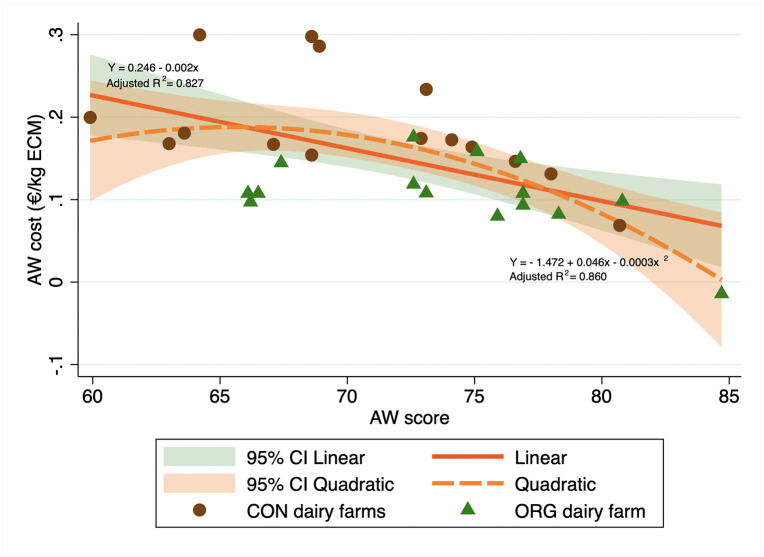
Scattered plot for conventional and organic farms; cost function (Linear vs. Quadratic) between AW score and AW cost.

This study employed a quadratic model to estimate AW costs, providing a better statistical fit and more accurately representing the case study data (Fig 5; Table 6). The model captures the non-linear relationship between AW scores and AW costs while controlling for other factors, which is important given the variability across farming systems. The case study shows that ORG farms generally have lower AW costs across the score spectrum, whereas CON farms exhibit greater variability and higher costs at lower scores (Fig 5). These patterns likely reflect differences in management practices, infrastructure, or resource allocation between farm types. However, the relationship between AW score and AW cost should be interpreted with caution, as it reflects associations rather than causal relationships due to the cross-sectional nature of the data. Coefficient estimates (β) in Table 6 indicate the strength and direction of each variable’s relationship with AW costs, with the intercept representing expected costs (€/kg ECM) when all other variables are zero. For instance, achieving an AW score of 86.17, where the model predicts zero additional cost, corresponds to approximately 6 €cents per kg ECM for ORG farms and 11 €cents for CON farms. These predicted costs are lower than the values reported in Table 4 (0.10 €/kg ECM for ORG and 0.20 €/kg ECM for CON) due to differences in model specification and adjustment for confounding variables. Overall, while the quadratic model highlights trends and farm-type differences, the results reflect associations and should not be interpreted as definitive causal effects or generalized to the entire region, underscoring the need for cautious interpretation of these findings.

**Table 6:** Estimating AW costs (€/kg ECM) for achieving AW standards with control other confounding factors affecting cost of production; β estimate of additional costs or cost savings; SE standard error.

Variable	Linear			Quadratic		
	Estimate (β)	S.E.	P-value	Estimate (β)	S.E.	P-value
AW score	−0.002	0.001	0.122	−0.002	0.001	**0.024**
AW score_square				−0.0003	0.0001	**0.018**
Management system (1: ORG, 0:CON)	−0.058	0.024	**0.027**	−0.046	0.023	**0.055**
Herd size (total of cows; LU)	−0.004	0.0008	**<0.000**	−0.002	0.001	**0.002**
Working time (h/LU/year)	−0.0001	0.0001	**0.032**	−0.0002	0.0001	**0.037**
Pastureland (ha)	−0.033	0.012	**0.014**	−0.037	0.011	**0.003**
Total cost (€/kg ECM/year)	0.207	0.039	**0.000**	0.203	0.035	**<0.000**
Intercept	0.246	0.095	**0.017**	0.291	0.087	**0.003**
N	30			30		
F-statistic	24.13			26.52		
F-value	<0.0000			<0.0000		
R^2^	0.8629			0.8941		
Adjusted R^2^	0.8271			0.8604		
The Breusch–Pagan test (P-value chi2)	0.7429			0.9084		
The Shapiro-Wilk test to residuals (P-value z)	0.88963			0.5256		

Bold value indicates significant different at 5%

## Discussion

### AW condition in Alpine dairy farms

This case study demonstrates that the total AW scores for both resource-based and animal-based indicators of dairy cow welfare differed between management systems, particularly in terms of water flow rate, skin alterations, and locomotion. Analysis of water provision revealed significant differences in water flow rates between the two farm types. All ORG farms met the required water flow rate of > 10 L/min, while only 11 out of 15 CON farms did so. As noted by Murphy [42] and Jensen and Vestergaard [43], restricted water access, such as fewer drinking opportunities or limited trough access, leads to increased drinking frequency and duration when water is available. However, this often results in reduced overall water intake, which can negatively affect both milk yield and AW. Our study complements the findings of Poulopoulou et al. [22] that water provision and space per cow are key factors limiting AW in Alpine dairy farms, irrespective of housing and management systems. These results may be attributed to local farmers’ lack of awareness regarding the importance of unrestricted access to clean drinking water. Additionally, most barns in the case study area, built in the 1980s, were constructed without consideration for AW, and space calculations excluded overall barn density.

Skin alterations are a major welfare concern for dairy cattle, as they can be painful and significantly affect the animals’ well-being [44]. In this study, the prevalence of integument alterations on the neck was similar across both ORG and CON farms. Tie-stalls were present in 20% of both farm types, and previous research by Katzenberger et al. [24] indicates that constant rubbing and chafing from collars in tie-stalls often cause hair loss. Additionally, the horizontal positioning of the tie-rail above the feed trough may contribute to the formation of swelling when cows push forward to reach feed. However, ORG farms showed a lower prevalence of skin alterations compared to CON farms, indicating higher welfare scores. This is likely due to more frequent access to pasture and better bedding practices [45]. Previous studies have linked skin integrity issues, particularly on the legs, to housing conditions and dairy breed differences [24]. These issues were often caused by hard stall bases, limited bedding materials, and inadequate resting surfaces [46]. Therefore, the prevalence of skin alterations in this study might be associated with a combination of housing conditions and management practices.

Locomotion difficulties were more prevalent in cows when moving than when standing (Table 3), which is consistent with previous research [21]. Locomotion scoring while moving tends to be more sensitive and accurate, especially in systems where cows have regular access to pasture or are housed in pens. This could explain the observed differences in lameness prevalence between farm types, as assessments in this study were conducted by trained researchers and veterinarians, ensuring rigorous reporting. Significant differences in locomotion assessments were observed between ORG and CON farms, both when standing and moving (Table 3). Studies from Europe and North America consistently report that ORG farms have a lower prevalence of lameness compared to CON farms. This is likely due to ORG regulations requiring pasture access, less intensive management, and smaller herd sizes, all factors associated with reduced risk of lameness [47,48]. Even limited access to pasture (as little as 2 hours per day) has been shown to reduce lameness by 1.5 to 2% for each additional hour of pasture access. Pasture provides a softer, cleaner surface, reducing claw horn lesions and trauma compared to hard indoor flooring, thereby supporting better hoof health and mobility [49,50]. Simmental cows, in particular, are better adapted to varying flooring and movement constraints compared to Holstein Friesian [46], which may further explain differences in lameness prevalence between farms. Regardless of management system, lameness was more closely associated with access to pasture, suggesting that increasing pasture access could help reduce lameness in dairy cows.

In general, the results of this study indicate that CON farms are more vulnerable to welfare challenges under the AW protocols and scoring systems used, with lower AW scores observed in farms utilizing tie-stalls and having limited pasture access (Fig 4). This aligns with previous findings that dairy cows’ welfare is influenced by the interaction between housing type and pasture access in South Tyrol, as assessed using the ClassyFarm protocol [46]. Although the total AW score was significantly higher for ORG farms compared to CON farms, the observed trend suggests that organic management may contribute to overall improvements in welfare, particularly regarding water flow, skin health, and locomotion. These findings imply that adopting organic practices could provide incremental welfare benefits, even when not all individual indicators reach statistical significance. Supporting this, studies using the Welfare Quality® protocol consistently report that ORG farms achieve higher welfare scores than CON farms [51]. Nonetheless, substantial variability within each farming system underscores the critical role of farm-specific management. Some CON farms attain high welfare scores when they voluntarily implement enhanced welfare practices, demonstrating that, although the type of farming system influences outcomes, individual farm management remains a decisive factor in determining animal welfare.

### Economic performance

Total production costs for both management systems (ORG and CON farms) in our study were higher compared to those reported in other alpine regions, such as Switzerland [52,53], excluding opportunity costs), Bavaria (Southern Germany) [54], and Austria [55]. This can primarily be attributed to smaller herd sizes and lower productivity. CON farms exhibited lower variability in milk yield and economic efficiency, while ORG farms showed more variability. The differences in economic efficiency may be explained by variations in productivity, husbandry systems, and the degree of management intensity (e.g., feeding practices, forage quality), all of which are influenced by geographic factors such as altitude and terrain steepness, as shown by Sturaro et al. [56]. Unlike previous studies (e.g., Berentsen et al. [57]), our study did not find significant differences in veterinary costs between CON and ORG farms. This may be due to both groups using the same dual-purpose cattle breed and having similar management practices, except for differences in feeding practices. ORG farms exhibited higher overall and operating costs compared to CON farms, but no significant differences were observed at return levels 1–3. The higher costs per kg of milk in ORG farms can be attributed to both lower milk production per farm and the higher costs of organic feedstuffs, particularly concentrated feed [57]. On the income side, the higher milk price paid for organic milk in South Tyrol (Table 5) helped compensate for the lower milk yield per cow, making milk price a crucial factor in adopting higher AW practices.

Concerning farm infrastructure, most dairy cows in the case study were housed in free-stall barns modernized in the 2000s but still retained dimensions from the 1980s. This likely explains why the barns do not meet the resource-related AG Rind criteria, as the reconstruction did not constitute a complete redesign to current standards, despite the farmers receiving subsidies for renovations. Consequently, the primary cost in this study is considered to be related to the need for barn reconstruction. Pasture access was less of a cost concern for ORG farms, as most already met the pasture criteria, resulting in no significant additional costs. In contrast, CON farms faced significant challenges in providing additional pasture access. Regardless of whether access was for a short or long period, increased pasture access often requires more alpine pastureland and infrastructure or, alternatively, a reduction in herd size, which sacrifices milk yield [58].

### Estimating cost of improving AW in achieving AW standards

In this study, we hypothesized that a higher AW score would be associated with lower AW costs, rather than higher AW costs leading to increased milk production costs. Our results support this hypothesis, as higher AW scores are indeed associated with lower AW costs, as shown in Table 6. This suggests that achieving higher AW standards does not necessarily incur additional costs for farmers, although the specific criteria or AW standard used may influence this outcome. To the best of our knowledge, this is the first study to explore the relationship between AW score and AW cost using empirical farm-level data. Previous research, such as the report by Tergast [10], found that higher AW standards led to increased AW costs, ranging from €0.20 to €0.10 per kg ECM for conventional dairy farms in Northwest Germany. Similarly, Vissers et al. [23] reported that ORG farms incurred an additional €0.02 per kg ECM, while CON farms paid €0.10 per kg ECM to meet AW standards. In contrast, our study found higher AW costs for both ORG and CON farms, due to differences in the AW standards to be achieved, economic conditions, barn constructions and implementing a grazing system in mountainous regions [36,46]. This case study underscores that farms with different initial conditions incur varying costs to achieve the same AW standards. We argue that aggregating CON and ORG farms to estimate the cost of improving AW based on milk production costs is methodologically questionable. Indeed, interpreting AW cost for ORG and CON farms has to be taken with care. Not only were there cases where some ORG sample farms incurred higher AW costs than comparable CON farms, but the degree of variation within the samples was also relatively high [59].

Despite the higher AW costs observed in our study, these costs remain below the willingness to pay (WTP) for improved welfare, as summarized in S2 Table. This suggests that farmers could potentially receive compensation for implementing higher AW standards through the milk price. However, the studies quantifying WTP for higher welfare milk in South Tyrol are limited. One relevant study by Busch et al. [60] reported that nearly 50% of South Tyrolean respondents were willing to pay a premium for locally produced, pasture-raised milk, which they perceived as more animal-friendly and healthier. This highlights the potential market for higher welfare milk, even though current certification schemes like the National Quality System for Animal Welfare (SQNBA) in Italy do not guarantee a higher milk price. Nevertheless, evidence from Zanon et al. [61] show a significant positive association between AW scores and milk sales per cow per year in small-scale mountain dairy farms, but this is due to improved cow performance, not price regulation, as higher AW scores could favour healthier cows and lower milk contamination. From a policy perspective, this research offers valuable insights for policymakers. However, more comprehensive data analysis is needed to ensure broader regional representation and to understand how subsidies or milk price premiums can help offset the additional costs incurred by farmers when adopting higher AW standards. Further complicating this picture, our study found that some ORG farms had lower AW scores despite higher AW costs. This observation suggests that the relationship between AW scores and AW costs is not always linear. More efficient dairy farms, particularly those with larger scales, may incur lower production costs while still achieving higher AW scores. This could be due to better management practices or economies of scale, as suggested by previous studies [62,63].

Theoretically, farmers make individual decisions about implementing AW practices, typically prioritizing low-cost and easy adjustments to minimize expenses while ensuring stable returns [64]. Assuming that AW criteria remain constant over time, the relationship between AW scores and AW costs could follow an inverted U-shaped curve (Fig 5). Initially, costs may rise gradually with low-effort modifications, such as staff training. These costs could then accelerate with more resource-intensive investments, like infrastructure upgrades, and eventually decline as fewer criteria need to be met. This model, however, may not be universally applicable, as factors such as farm size, milk yield, and farm efficiency (Fig 4) can significantly alter the shape of the cost curve. In practice, farm decisions on adopting higher AW practices vary significantly. Some farmers are driven by ethical considerations, market premiums for higher-welfare products, or regulatory pressures, rather than solely by cost minimization [65,66]. Bergschmidt and Schwarze [67] reported that farmers in Germany’s ‘Summer Grazing’ program increased grazing area per animal and improved husbandry conditions, although funding was insufficient to cover the additional costs. Further, Bergschmidt et al. [68] suggested that in AW support measures, resource-based and animal-based indicators are necessary to address all dimensions of animal welfare.

### Future developments and limitations of the case study in monetizing AW methods

This study highlights several areas for future development in monetizing AW methods, with particular attention to the weighting and aggregation of indicators, as these choices directly influence AW scores and their interpretation. To refine the AW score, it is essential to account for both the severity of animal-based indicators and their economic implications, allowing each component to be appropriately weighted. Some protocols assign equal weights for simplicity (e.g., Welfare Quality®, Classyfarm) [69,70], whereas others apply differential weights based on empirical data, perceived impact, or ethical considerations [5]. In the present case study, a ratio scale is used to reflect that higher AW scores in the initial farm condition correspond to lower AW costs, with greater weights on more impactful indicators amplifying economic significance. Overall AW scores are aggregated by summing weighted indicator scores, ensuring that no indicator fully compensates for another. This approach aligns with Dusel and Wieck [5], who argue that weighting should prevent compensation across indicators, and addresses Fischer’s [71] concern that ordinal scale assessments inadequately capture welfare effects on affected animals. Decisions regarding weighting and aggregation could reflect local production practices, species-specific welfare priorities, and economic or regulatory considerations, thereby influencing AW scores, estimated costs, and cross-farm comparisons. Future research will further refine indicator weights and aggregation procedures, guided by consensus among key stakeholders, including researchers, policy makers, farmers, consumers, and industry representatives, to ensure that the AW scoring system accurately represents both animal welfare severity and associated economic outcomes.

Another important development is the establishment of clear AW standards or criteria levels. Defining specific targets or benchmarks will provide farmers with measurable goals to meet. For instance, in Germany, dairy farmers are incentivized by cooperatives when they meet specific AW criteria, such as QM+ or QM++ (S2 Table). Additionally, farmers may receive higher milk prices depending on the level of AW criteria achieved, as indicated by the animal welfare labelling initiative [72]. Further, the aggregation of AW scores should not merely categorize welfare on a simple best-to-worst continuum. Rather, it should account for the specific targets and conditions that policymakers and consumers are willing to support financially. This approach will ensure a more comprehensive and economically relevant evaluation of AW standards. To sum up, this case study presents conceptual methods for making informed decisions about whether the incentives or subsidies farmers receive sufficiently offset the costs incurred to meet certain standards.

This study has several limitations that should be considered when interpreting the results. The sample size was limited to 30 dairy farms, making the findings specific to this case study. The analysis relied on lying space indicators to identify which farmers should reconstruct their barns, without considering other factors such as barn layout, existing space, and herd size. Furthermore, the focus on herd size and pastureland excluded other factors, such as housing systems, which may impact cost estimates. These limitations, along with the sample size and variability, could affect the shape and slope of the cost curves, potentially leading to different results. Additionally, estimating costs related to animal-based indicators is complex, as each parameter has distinct cost implications, underscoring the need for collaboration between animal welfare scientists and economists to better integrate animal welfare into policy analysis. Despite these limitations, this study provides valuable insights into the economic implications of AW improvements and lays the foundation for future research.

## Conclusion

This case study estimates the economic implications of improving animal welfare (AW) in Alpine dairy farming, focusing on Simmental cattle in both conventional (CON) and organic (ORG) farming systems in South Tyrol, Italy. While the study found that ORG farms generally achieve higher AW scores, they also incur higher production costs due to the dilution effect caused by lower milk production. In contrast, CON farms showed higher AW costs per unit of milk produced, primarily due to the additional resources required to meet welfare standards. This study also introduces a novel concept for calculating AW costs, addressing methodological concerns in previous studies that used milk production costs as a proxy. However, it is important to note that these findings are based on a limited sample size of 30 farms, and results may not be broadly generalizable to all farming systems or Alpine region. The analysis revealed a nonlinear relationship between AW scores and AW costs, suggesting that higher animal welfare in initial conditions could lead to cost savings, particularly in larger and more efficient farms. Indeed, variability across farms, including factors such as farm size, management practices, and regional differences, should be considered when interpreting these results. Further, the importance of setting clear AW standards and scoring systems that reflect both the welfare impacts on animals and the economic feasibility for farmers is emphasized. It is argued that the aggregation of AW scores should not simply rank welfare along a basic best-to-worst spectrum but instead consider the specific goals and conditions that policymakers and consumers are willing to financially support. Potential policies could include subsidies for farms transitioning to higher welfare standards, financial support for infrastructure upgrades, or higher milk prices for those meeting AW criteria. Future studies should involve longitudinal data collection on different breeds (other dual-purpose or milk-specialized), AW assessments, dairy cow performance, and farm economic outcomes to enable rigorous calculation of additional costs and potential benefits. It is also suggested that cluster analyses be conducted across different farming conditions (e.g., housing type, grazing access, and bedding materials) using larger datasets to obtain a clearer understanding of how AW cost behavior varies across diverse farm types and conditions.

## Supporting information

S1 Table Farm characteristics. (DOCX)

S2 Table Method of monetize and milk cost AW improvements [73–87]. (DOCX)
