# Supplementary material for: From theory to practice: A case study on estimating the costs of improving animal welfare on Simmental alpine dairy farms
Source: PLoS One. 2026 Jul 31;21(7):e0343380. doi: 10.1371/journal.pone.0343380 (PMC13426955; doi:10.1371/journal.pone.0343380)
Supplement: S1 Table — (DOCX) [file pone.0343380.s001.docx]

**S1 Table.** **Farm characteristics with Mean, Median, Minimum (Min), and Maximum (Max) values, as well as Standard Deviation (SD) for conventional (CON) and organic (ORG) farms (n = 15, respectively).**

| **Farm characteristic** |  | **CON** | | | | |  |  | **ORG** | | | | |
| --- | --- | --- | --- | --- | --- | --- | --- | --- | --- | --- | --- | --- | --- |
|  | **N** | **Mean** | **Median** | **Min** | **Max** | **SD** |  | **N** | **Mean** | **Median** | **Min** | **Max** | **SD** |
| Elevation (m a.s.l.) |  | 1,148 | 1,117 | 836 | 1,450 | 189 |  |  | 1,218 | 1,302 | 650 | 1,500 | 246 |
| No dairy cows |  | 17.5 | 17.0 | 10.0 | 30.0 | 5.2 |  |  | 16.1 | 16.0 | 3.0 | 36.0 | 7.9 |
| No calves (< 6 months) |  | 3.7 | 4.0 | 0.0 | 10.0 | 2.5 |  |  | 2.4 | 2.0 | 0.0 | 6.0 | 1.7 |
| No calves (6-24 months) |  | 4.7 | 5.0 | 0.0 | 10.0 | 2.9 |  |  | 4.0 | 3.0 | 0.0 | 10.0 | 2.8 |
| No heifers |  | 0.0 | 0.0 | 0.0 | 0.0 | 0.0 |  |  | 0.1 | 0.0 | 0.0 | 1.0 | 0.4 |
| No bulls |  | 0.3 | 0.0 | 0.0 | 3.0 | 0.9 |  |  | 0.1 | 0.0 | 0.0 | 1.0 | 0.4 |
| Permanent grassland (ha) alpine pastures not included |  | 12.4 | 11.8 | 5.1 | 20.5 | 4.9 |  |  | 12.4 | 13.0 | 2.6 | 24.0 | 5.3 |
| Alpine pastures (ha) |  | 5.7 | 2.0 | 0.0 | 19.0 | 7.0 |  |  | 1.7 | 0.0 | 0.0 | 16.0 | 4.2 |
| Arable land (ha) |  | 0.1 | 0.0 | 0.0 | 1.0 | 0.3 |  |  | 0.0 | 0.0 | 0.0 | 0.6 | 0.2 |
| Grassland yields (kg DM * ha^-1^) including pasturage and alpine pastures |  | 5,180 | 5,015 | 2,931 | 7,000 | 1,567 |  |  | 5,203 | 5,250 | 2,623 | 6,446 | 961 |
| Number of cuts |  | 3.14 | - | 1 | 6 | 1.08 |  |  | 2.0 | - | 1 | 3 | 0.72 |
| Stocking rate (LU ha^-1^) |  | 1.53 | 1,60 | 0,86 | 2,10 | 0,37 |  |  | 1.31 | 1,30 | 0,85 | 1,66 | 0,28 |
| Days of pasture dairy cows (days year^-1^) |  | 25.3 | 30 | 0 | 60 | 25.7 |  |  | 201.3 | 120 | 280 | 200 | 36.9 |
| Milk yield (kg ECM * animal, year ^-1^) |  | 9,505 | 9,471 | 8,031 | 10,817 | 912 |  |  | 6,540 | 6,611 | 3,454 | 9,294 | 1,336 |
| Free-stall barns | 11 |  |  |  |  |  |  | 13 |  |  |  |  |  |
| Tie-stall barns | 4 |  |  |  |  |  |  | 2 |  |  |  |  |  |
| Number of trough drinkers |  | 0.9 | - | 0 | 2 | 0.68 |  |  | 1.4 | - | 0 | 3 | 0.88 |
| Number of bowl drinkers |  | 3.5 | - | 0 | 11 | 3.50 |  |  | 1.73 | - | 0 | 5 | 1.65 |
| Water flow rate ≥ 10 l*min-1 | 11 |  |  |  |  |  |  | 15 |  |  |  |  |  |
| Water flow rate ≤ 10 l*min-1 | 3 |  |  |  |  |  |  | 0 |  |  |  |  |  |
| Cleanieness drinkers (yes) | 13 |  |  |  |  |  |  | 14 |  |  |  |  |  |
| Cleanieness drinkers (no) | 2 |  |  |  |  |  |  | 1 |  |  |  |  |  |
