# Supplementary material for: From theory to practice: A case study on estimating the costs of improving animal welfare on Simmental alpine dairy farms
Source: PLoS One. 2026 Jul 31;21(7):e0343380. doi: 10.1371/journal.pone.0343380 (PMC13426955; doi:10.1371/journal.pone.0343380)
Supplement: S2 Table — (DOCX) [file pone.0343380.s002.docx]

**S2 Table. Method of monetize and milk cost AW improvements.**

| Country | Year | Methods | Conventional | | | | | Organic | | | Authors |
| --- | --- | --- | --- | --- | --- | --- | --- | --- | --- | --- | --- |
| PL, ES, UK, SE, IT, MK, NL, DE | 2010 | Cost-Benefit Analysis (CBA) | €/litre  DE  PL  ES  UK  SE  IT  MK  NL | Moderate  +0.03  n.a.  n.a.  +0.0001  +0.01  +0.01  +0.01  +0.01 | | | Premium  –0.008  +0.01  n.a.  +0.0001  +0.02  +0.02  +0.01  –0.008 | not available (n.a.) | | | [7] |
| Germany | 2010 | Willingness-to-pasy (WTP) | n.a. | | | | | 0.58 €/liter more, than Conventional | | | [11] |
| FI, DE, IE, NL, PL, AR, NZ | 2010 | Partial Budgeting cost of compliance with EU legislation | €/kg  DE31  DE95  DE650  FI25  FI69  IE48  IE115  NL76  PL15  PL65  AR170  AR400  NZ974 | Welfare  0.0023  0.0009  0.0027  0.0000  0.0000  0.0000  0.0000  0.0000  0.0000  0.0000  0.0000  0.0000  0.0000 | | | Health  0.0042  0.0027  0.0019  0.0110  0.0094  0.0034  0.0037  0.0052  0.0018  0.0009  0.0015  0.0013  0.0019 | n.a. | | | [8] |
| Germany | 2013 | WTP | 0.096 €/kg more, for pasture milk | | | | | n.a. | | | [73] |
| Italy | 2013 | CBA | 91.16–182.32 €/LU for acheiving the standard animal welfare | | | | |  | | | [74] |
| Germany | 2014 | WTP | Based price: 0.69 €/kg | | | | | 0.27 €/kg more, than Conventional | | | [75] |
| Italy | 2014 | CBA | 8.29–27.02 €/LU for increasing animal welfare by 10% of Welfare Quality® protocols | | | | | 14.52 €/LU for increasing animal welfare by 10% of WQ® protocols | | | [76] |
| Germany | 2015 | CBA | 3%/338 million€, lower=2%/225, upper=5%/451 from the based cost | | | | | n.a. | | | [9] |
| Germany | 2015 | WTP | 39% from the based price | | | | | n.a. | | | [2] |
| Germany | 2015 | WTP | - 0.50 €/litre more, for pasture milk certified - 0.38 €/litre more, for pasture milk marketing ads claim | | | | | n.a. | | | [77] |
| Germany | 2016 | CBA | 0.10 €/kg | | | | | n.a. | | | [78] |
| Germany | 2017 | WTP | Based price: 0.88 €/kg | | | | | 0.48 €/kg more, than Conventional | | | [79] |
| Germany | 2017 | WTP | 0.24 €/litre more, from the based price | | | | | n.a. | | | [80] |
| Italy | 2017 | WTP | n.a. | | | | | 0.25 €/liter more, than Conventional | | | [12] |
| 8 European countries | 2018 | WTP | €/litre  Organic (OR)  Grazing (GR)  Tie-stall  Compost bedded  Artificial floor | | | | GR  0.21  0.29  0.41  0.55  0.55 | €/litre  Organic  Grazing  Tie-stall  Compost bedded  Artificial floor | OR  0.34  0.18  0.34  0.11  0.24 | OR&GR  0.32  0.30  0.08  0.28  0.40 | [81] |
| Germany | 2019 | Calculation data  German Animal Welfare Federation programme | €/kg ECM  Entry  Premium | | | | 0.0228  0.0264 | n.a. | | | [82] |
| Germany | 2019 | CBA | 0.10 €/kg ECM | | | | | 0.02 € per kg ECM | | | [23] |
| Germany | 2021 | CBA | €/kg ECM  Level 1  Level 2  Level 3 | | | | 0.0392  0.0415  0.0749 | n.a. | | | [83] |
| Germany | 2022 | CBA | €/kg  Level 2  Level 3  Level 4 | Tie stall    Old barn  Lifespan  Pasture | | | 0.0991  0.0428  0.04–0.07  0.06–0.09 | n.a. | | | [84] |
| Germany | 2022 | Calculation data  QM+ Incentive programme/CBA | 0.012 €/kg ECM | | | | | n.a. | | | [85] |
| Germany | 2022 | Expert interview | €/kg ECM  QM+  QM++ | | 0.0670 – 0.1360  0.0060 – 0.0680 | | | n.a. | | | [86] |
| Germany | 2022 | Calculation data | €/kg ECM  German AW program  DLG  QM  Pro Weideland | Entry  Premium  Bronz  Silver  Gold  QM+  QM++ | | | 0.0450  0.0568  0.0100  0.0393  0.0570  0.0091  0.0386  0.0497 | n.a. | | | [3] |
| Germany | 2022 | CBA  PraeRi-project Animal Welfare programme | €/kg ECM  North  East  South | | | 0.1197  0.1063  0.1633 | | n.a. | | | [87] |
| Germany | 2022 | CBA | 0.025–0.10 €/kg ECM | | | | | n.a. | | | [10] |
| Switzerland | 2022 | WTP | n.a. | | | | | 0.30 €/liter more, than Conventional | | | [13] |
