# Supplementary material for: From theory to practice: A case study on estimating the costs of improving animal welfare on Simmental alpine dairy farms
Source: PLoS One. 2026 Jul 31;21(7):e0343380. doi: 10.1371/journal.pone.0343380 (PMC13426955; doi:10.1371/journal.pone.0343380)
Supplement: Attachment — Submitted filename: Reviewer.pdf [file pone.0343380.s003.pdf]

Line 40 : In the keyword section, there is a term appeared "Dual-purpose breeds" but the manuscript rarely discusses the dual-purpose characteristic of Simmental. The whole paper focus is on dairy production. If this keyword stays, there should be at least a paragraph somewhere (I recommend Introduction) and explain why the dual-purpose nature imporant for AW economics.

Line 75: *The transition from ordinal scales (Katzenberger et al. [20]) to ratio scales (Poulopoulou et al. [21]*) is mentioned in one sentence. Please explain it a little bit because it won't be easy for some readers who are not familiar with these kind of protocols. A couple of sentences would suffice.

Line 151: "Locommotion" – typo. Should be "Locomotion." This appears in Table 1 and Table 3 (Line 273).

Line 234: Use Vissers et al. instead of Visser et al.

Line 272-276: In Table 3, The total AW score difference between CON and ORG is significant at $p = 0.046$ that just under the 0.05 threshold. Meanwhile, Table 3 reports tests for many individual indicators, most of which are not significant. With this number of comparisons, the chance of a borderline result increases. I am not asking for a formal correction, but some caution in the interpretation would be appropriate.

Line 323: In Table 4, Some values are extremely small (e.g., -0.0001 for AI costs in ORG). Reporting to four decimal places for such numbers gives a misleading impression of precision. Consider rounding, please.

Line 368: The quadratic model includes AW score and AW score^2 (AW score_square – Table 6), which are naturally correlated. This is a known issue in polynomial regressions. Did the authors center the AW score variable before squaring? If not, multicollinearity could inflate standard errors. Reporting VIF values would clarify this.

Line 625-627 The reference says OIE (2015), but the annual report shows 2013. If it is 2013, reference should be updated else not.
