# Supplementary material for: From theory to practice: A case study on estimating the costs of improving animal welfare on Simmental alpine dairy farms
Source: PLoS One. 2026 Jul 31;21(7):e0343380. doi: 10.1371/journal.pone.0343380 (PMC13426955; doi:10.1371/journal.pone.0343380)
Supplement: Attachment — Submitted filename: Response to reviewers.docx [file pone.0343380.s005.docx]

Dear Yasin ALTAY, Ph. D.,

We appreciate the opportunity to resubmit our manuscript, titled *“From Theory to Practice: A Case Study on Estimating the Costs of Improving Animal Welfare on Simmental Alpine Dairy Farms,”* for consideration in PLOS ONE. We have carefully revised the manuscript based on the reviewer’s comments. The main revisions we have made include:

1. **Clarification of Statistical Methods**: We have emphasized the non-causal nature of the associations between animal welfare (AW) scores and AW costs, as suggested by the reviewer. We have updated the manuscript to clarify that the findings reflect associations rather than definitive causal relationships due to the cross-sectional nature of the data.
2. **Explanation of Dual-Purpose Breeds**: Following the reviewer’s suggestion, we have added a section explaining the importance of dual-purpose breeds, specifically Simmental, in alpine regions. This explanation is now included in the Materials and Methods section, as it is essential to the economic resilience of farming in such challenging environments.
3. **Typographical and Reference Corrections**: We have corrected several minor typographical errors (e.g., "Locommotion" to "Locomotion" and "Vissers" to "Visser") and updated references where necessary.
4. **Additional Clarification on AW Scoring and Cost Calculation**: We have provided more detail on the weighting and aggregation of AW indicators, as this directly influences the interpretation of AW scores and their economic implications.
5. **Improved Discussion and Conclusion**: The interpretation of our findings has been moderated to better reflect the limitations of the study design and the small sample size. We also emphasize the need for caution in generalizing these findings to other farming systems.
6. **Data Availability and Funders’ Role**: We have included the required statement regarding the funders' role: *“The funders had no role in study design, data collection and analysis, decision to publish, or preparation of the manuscript.”* We also addressed the reviewer’s comment on the data availability statement, clarifying that the data is available upon request due to privacy concerns.

We believe that these revisions address the reviewer’s concerns and have significantly improved the manuscript. We look forward to your feedback and hope for a positive consideration of our revised submission.

Sincerely,
Dr. Thomas Zanon

| Line | Revision |
| --- | --- |
| - | **Please ensure that your manuscript meets PLOS ONE's style requirements, including those for file naming.** The PLOS ONE style templates can be found at  https://journals.plos.org/plosone/s/file?id=wjVg/PLOSOne_formatting_sample_main_body.pdf and https://journals.plos.org/plosone/s/file?id=ba62/PLOSOne_formatting_sample_title_authors_affiliations.pdf  We have reviewed the PLOS ONE style requirements using the template provided in our manuscript. |
| 645 | **Your ethics statement should only appear in the Methods section of your manuscript. If your ethics statement is written in any section besides the Methods, please delete it from any other section.**  **If there are ethical or legal restrictions on sharing a de-identified data set, please explain them in detail (e.g., data contain potentially identifying or sensitive patient information, data are owned by a third-party organization, etc.) and who has imposed them (e.g., a Research Ethics Committee or Institutional Review Board, etc.). Please also provide contact information for a data access committee, ethics committee, or other institutional body to which data requests may be sent.**  **If your data cannot be made publicly available for ethical or legal reasons (e.g., public availability would compromise patient privacy), please explain your reasons on resubmission and your exemption request will be escalated for approval.**  **We note that you have indicated that there are restrictions to data sharing for this study. For studies involving human research participant data or other sensitive data, we encourage authors to share de-identified or anonymized data. However, when data cannot be publicly shared for ethical reasons, we allow authors to make their data sets available upon request.**  We appreciate the publisher's concern regarding data sharing. As the data utilized in this study was collected from farmers, privacy considerations are of utmost importance. While the data has been de-identified, it still contains potentially sensitive information, thereby precluding its unrestricted sharing. Access to the dataset is governed by ethical and privacy guidelines informally established by our institution and the Association for Animal Breeding in South Tyrol (Vereinigung der Südtiroler Tierzuchtverbände). To request access, interested parties are encouraged to contact the Data Access Person at [TZ]. All requests will be reviewed in accordance with applicable ethical and legal standards. |
| 660 | **Please state what role the funders took in the study. If the funders had no role, please state: "The funders had no role in study design, data collection and analysis, decision to publish, or preparation of the manuscript."**  **If this statement is not correct you must amend it as needed.**  **Please include this amended Role of Funder statement in your cover letter; we will change the online submission form on your behalf.**  We have added the statement on sub-section Funding. |
| 973 | [**Please include captions for your Supporting Information files at the end of your manuscript, and update any in-text citations to match accordingly. Please see our Supporting Information guidelines for more information: http://journals.plos.org/plosone/s/supporting-information.**](https://eur03.safelinks.protection.outlook.com/?url=http%3A%2F%2Ftrack.editorialmanager.com%2FCL0%2Fhttp%3A%252F%252Fjournals.plos.org%252Fplosone%252Fs%252Fsupporting-information.%2F1%2F010f019d6af87b1a-f1ad5aa8-e7d2-4920-99a7-a85850c7fdb8-000000%2Ff0NWRbXHwxV9GzY7zuCNGztEKoZTJ4wbMcdRrRTBXH0%3D258&data=05%7C02%7CAgung.Triatmojo%40student.unibz.it%7Cd18c37d1b8d04f9449cc08de95338c6a%7C9251326703e3401a80d4c58ed6674e3b%7C0%7C0%7C639112245798263907%7CUnknown%7CTWFpbGZsb3d8eyJFbXB0eU1hcGkiOnRydWUsIlYiOiIwLjAuMDAwMCIsIlAiOiJXaW4zMiIsIkFOIjoiTWFpbCIsIldUIjoyfQ%3D%3D%7C0%7C%7C%7C&sdata=JCU5f1MOJGHLWs%2BvrSUDC3J1O1JABjRV5VC2Qrp%2FTkE%3D&reserved=0)  We have added the Supporting information at the end pf our manuscript. |
| - | **We notice that your supplementary [tables] are included in the manuscript file. Please remove them and upload them with the file type 'Supporting Information'. Please ensure that each Supporting Information file has a legend listed in the manuscript after the references list.**  We have deleted the supplementary (tables) |
| - | **If the reviewer comments include a recommendation to cite specific previously published works, please review and evaluate these publications to determine whether they are relevant and should be cited. There is no requirement to cite these works unless the editor has indicated otherwise.**  Thank you for the explanation |
| Reviewer 1 | |
| 40  103  617 | **In the keyword section, there is a term appeared "Dual-purpose breeds" but the manuscript rarely discusses the dual-purpose characteristic of Simmental. The whole paper focus is on dairy production. If this keyword stays, there should be at least a paragraph somewhere (I recommend Introduction) and explain why the dual-purpose nature imporant for AW economics.**  We appreciate the reviewer’s suggestion regarding the keyword "dual-purpose breeds." This term is essential as breeds like Simmental are typical in alpine regions, where conditions differ significantly from arable areas with milk-specialized breeds. Dual-purpose breeds offer economic resilience in such challenging environments, making this aspect a key novelty of our study. Although the reviewer recommended placing the explanation in the Introduction, we have included it in the Materials and Methods section to maintain clarity and conciseness. This section provides readers with a clear understanding of why dual-purpose breeds are crucial for alpine regions.  Materials and methods  The study focused exclusively on farms that raised purebred Simmental cattle, as this breed is commonly used in the region and is particularly suitable for small-scale alpine dairy farming [28]. In the Alps, mixed dairy–beef or dual-purpose breeds like Simmental are still widely used due to their adaptability to the challenging production conditions of these farms [29]. Further, dual-purpose breeds can remain economically competitive in mountainous regions due to the value of calves, cull cows, and subsidies, even though they produce lower milk yields compared to milk-specialized breeds [28].  Conclusion  Future studies should conduct longitudinal data collection on different breeds (other dual-purpose or milk-specialized), AW assessments, dairy cow performance, and farm economic outcomes to enable rigorous calculation of additional costs and potential benefits. It is also suggested that cluster analyses be conducted across different farming conditions (e.g., housing type, grazing access, and bedding materials) using larger datasets, to obtain a clearer understanding of how AW cost behavior varies. |
| 75  72 | **The transition from ordinal scales (Katzenberger et al. [20]) to ratio scales (Poulopoulou et al. [21]) is mentioned in one sentence. Please explain it a little bit because it won’t be easy for some readers who are not familiar with these kind of protocols. A couple of sentences would suffice.**  Several AW protocols (structured guidelines for assessing and ensuring animal well-being through indicators and interventions) have been developed to integrate both human and animal perspectives [18]. These protocols may use qualitative nominal or ordinal scales to classify indicators into categories (e.g., presence/absence or insufficient/acceptable/excellent), while quantitative ratio scales assign numerical values to measurable factors for more precise assessment. For example, the Welfare Quality® Protocol is an ordinal-scale framework in Europe, while Italy's ClassyFarm system also evaluates ordinal-scale animal welfare in dairy farming, focusing on biosecurity, farm management, animal-based measures, and hazard alarm systems [19,20]. However, these protocols partly lack clear thresholds for interpreting welfare scores, particularly in smaller herds where scores may be skewed [20]. Recent advancements have moved from qualitative nominal and ordinal scales (e.g., Katzenberger et al. [21]) to quantitative ratio scales, such as the work by Poulopoulou et al. [22], which assigns proportional scores to specific AW indicators. |
| 151 | **"Locommotion" – typo. Should be "Locomotion." This appears in Table 1 and Table 3 (Line 273).**  We have corrected the typo. Thank you for your careful attention to detail. |
| 234 | **Use Vissers et al. instead of Visser et al.**  We have corrected the typo in Figure 2. |
| 272-276  313  444 | **In Table 3, The total AW score difference between CON and ORG is significant at p = 0.046 that just under the 0.05 threshold. Meanwhile, Table 3 reports tests for many individual indicators, most of which are not significant. With this number of comparisons, the chance of a borderline result increases. I am not asking for a formal correction, but some caution in the interpretation would be appropriate.**  Result  Overall, the total AW score was significantly higher in ORG farms (73.99) compared to CON farms (70.28), with a p-value of 0.046. However, most individual indicators did not reach significance. Given the number of comparisons, this borderline significance should be interpreted with caution.  Discussion  In general, the results of this study indicate that CON farms are more vulnerable to welfare challenges under the AW protocols and scoring systems used, with lower AW scores observed in farms utilizing tie-stalls and having limited pasture access (Fig 4). This aligns with previous findings that dairy cows' welfare is influenced by the interaction between housing type and pasture access in South Tyrol, as assessed using the ClassyFarm protocol [46]. Although the total AW score was significantly higher for ORG farms compared to CON farms, the observed trend suggests that organic management may contribute to overall improvements in welfare, particularly regarding water flow, skin health, and locomotion. These findings imply that adopting organic practices could provide incremental welfare benefits, even when not all individual indicators reach statistical significance. Supporting this, studies using the Welfare Quality® protocol consistently report that ORG farms achieve higher welfare scores than CON farms [51]. Nonetheless, substantial variability within each farming system underscores the critical role of farm-specific management. Some CON farms attain high welfare scores when they voluntarily implement enhanced welfare practices, demonstrating that, although the type of farming system influences outcomes, individual farm management remains a decisive factor in determining animal welfare. |
| 323  345 | **In Table 4, Some values are extremely small (e.g., -0.0001 for AI costs in ORG). Reporting to four decimal places for such numbers gives a misleading impression of precision. Consider rounding, please.**  To avoid giving a misleading impression of precision in the costs or cost savings reported in Table 4, the values were not rounded. Instead, they were converted from €/kg ECM/year to € cents/kg ECM/year, as rounding values of 0.0001 to two or three decimals could introduce additional bias. |
| 368 | **The quadratic model includes AW score and AW score² (AW score_square – Table 6), which are naturally correlated. This is a known issue in polynomial regressions. Did the authors center the AW score variable before squaring? If not, multicollinearity could inflate standard errors. Reporting VIF values would clarify this.**  Thank you for your question. We have centered the AW_score variable before squaring it and have checked for multicollinearity. The VIF values are all below 10. A comparison of VIF values before and after centering the AW_score variable is provided in the end of this file. |
| 625-627  668 | **The reference says OIE (2015), but the annual report shows 2013. If it is 2013, reference should be updated else not.**  We have corrected the reference year. |
|  |  |
| Reviewer 2 | |
|  | **The manuscript addresses a relevant and timely topic by integrating animal welfare (AW) assessment with economic evaluation at the farm level. The combination of resource-based and animal-based indicators, along with the attempt to model AW costs, provides useful insights and contributes to ongoing discussions on the economic implications of improving animal welfare in dairy systems. Overall, the study is generally technically sound, and the analyses are appropriate. The manuscript is well written and well structured. However, several points should be clarified or slightly revised to improve transparency and interpretation.** |
| 369 | **While the statistical methods are appropriate, the interpretation of the relationship between AW score and AW cost could be presented more cautiously. Given the cross-sectional nature of the data, it would be helpful to emphasize that the findings reflect associations rather than causal relationships.**  This study employed a quadratic model to estimate AW costs, providing a better statistical fit and more accurately representing the case study data (Fig 5; Table 6). The model captures the non-linear relationship between AW scores and AW costs while controlling for other factors, which is important given the variability across farming systems. The case study shows that ORG farms generally have lower AW costs across the score spectrum, whereas CON farms exhibit greater variability and higher costs at lower scores (Fig 5). These patterns likely reflect differences in management practices, infrastructure, or resource allocation between farm types. However, the relationship between AW score and AW cost should be interpreted with caution, as it reflects associations rather than causal relationships due to the cross-sectional nature of the data. Coefficient estimates (β) in Table 6 indicate the strength and direction of each variable’s relationship with AW costs, with the intercept representing expected costs (€/kg ECM) when all other variables are zero. For instance, achieving an AW score of 86.17, where the model predicts zero additional cost, corresponds to approximately 6 €cents per kg ECM for ORG farms and 11 €cents for CON farms. These predicted costs are lower than the values reported in Table 4 (0.10 €/kg ECM for ORG and 0.20 €/kg ECM for CON) due to differences in model specification and adjustment for confounding variables. Overall, while the quadratic model highlights trends and farm-type differences, the results reflect associations and should not be interpreted as definitive causal effects or generalized to the entire region, underscoring the need for cautious interpretation of these findings. |
| 551 | **The AW scoring system would benefit from a brief additional clarification regarding the weighting and aggregation of indicators, as these choices may influence the results.**  This study highlights several areas for future development in monetizing AW methods, with particular attention to the weighting and aggregation of indicators, as these choices directly influence AW scores and their interpretation. To refine the AW score, it is essential to account for both the severity of animal-based indicators and their economic implications, allowing each component to be appropriately weighted. Some protocols assign equal weights for simplicity (e.g., Welfare Quality®, Classyfarm) [69,70], whereas others apply differential weights based on empirical data, perceived impact, or ethical considerations [5]. In the present case study, a ratio scale is used to reflect that higher AW scores in the initial farm condition correspond to lower AW costs, with greater weights on more impactful indicators amplifying economic significance. Overall AW scores are aggregated by summing weighted indicator scores, ensuring that no indicator fully compensates for another. This approach aligns with Dusel and Wieck [5], who argue that weighting should prevent compensation across indicators, and addresses Fischer’s [71] concern that ordinal scale assessments inadequately capture welfare effects on affected animals. Decisions regarding weighting and aggregation could reflect local production practices, species-specific welfare priorities, and economic or regulatory considerations, thereby influencing AW scores, estimated costs, and cross-farm comparisons. Future research will further refine indicator weights and aggregation procedures, guided by consensus among key stakeholders, including researchers, policy makers, farmers, consumers, and industry representatives, to ensure that the AW scoring system accurately represents both animal welfare severity and associated economic outcomes. |
| 220 | **Some economic assumptions (e.g., assumed milk yield reductions under grazing scenarios) could be more clearly justified or discussed in terms of potential variability across farms.**  We have clarified the economic assumptions regarding milk yield reductions under grazing scenarios by referencing focus group observations from Tergast et al. [10] and comparative studies [35, 36].  This study followed focus group observations from Tergast et al., [10], which indicate that the improving grazing access (120 days/6 hours per day; Table 2) initially increases milk yield at the start of pasture access, but this yield gradually declines over the grazing season, resulting in an overall reduction of approximately 1,000 kg ECM per cow per year. This pattern aligns with performance changes observed when farms transition from summer grazing to year-round housing and is consistent with comparative studies on pasture versus barn systems [35,36]. However, it is acknowledged that the extent of yield reductions may vary across farms depending on factors such as farm management practices, cow breed, pasture quality, and climate conditions. These factors represent limitations of the case study, as the assumptions may not be universally applicable to all farm types. |
| 597 | **The conclusion section is generally well written; however, the interpretation of the findings could be slightly moderated to better reflect the limitations of the study design and sample size.**  This case study estimates the economic implications of improving animal welfare (AW) in Alpine dairy farming, focusing on Simmental cattle in both conventional (CON) and organic (ORG) farming systems in South Tyrol, Italy. While the study found that ORG farms generally achieve higher AW scores, they also incur higher production costs due to the dilution effect caused by lower milk production. In contrast, CON farms showed higher AW costs per unit of milk produced, primarily due to the additional resources required to meet welfare standards. This study also introduces a novel concept for calculating AW costs, addressing methodological concerns in previous studies that used milk production costs as a proxy. However, it is important to note that these findings are based on a limited sample size of 30 farms, and results may not be broadly generalizable to all farming systems or Alpine region. The analysis revealed a nonlinear relationship between AW scores and AW costs, suggesting that higher animal welfare in initial conditions could lead to cost savings, particularly in larger and more efficient farms. Indeed, variability across farms, including factors such as farm size, management practices, and regional differences, should be considered when interpreting these results. Further, the importance of setting clear AW standards and scoring systems that reflect both the welfare impacts on animals and the economic feasibility for farmers is emphasized. It is argued that the aggregation of AW scores should not simply rank welfare along a basic best-to-worst spectrum but instead consider the specific goals and conditions that policymakers and consumers are willing to financially support. Potential policies could include subsidies for farms transitioning to higher welfare standards, financial support for infrastructure upgrades, or higher milk prices for those meeting AW criteria. Future studies should involve longitudinal data collection on different breeds (other dual-purpose or milk-specialized), AW assessments, dairy cow performance, and farm economic outcomes to enable rigorous calculation of additional costs and potential benefits. It is also suggested that cluster analyses be conducted across different farming conditions (e.g., housing type, grazing access, and bedding materials) using larger datasets to obtain a clearer understanding of how AW cost behavior varies across diverse farm types and conditions. |
| - | **Finally, the Data Availability Statement should be revised to fully comply with PLOS ONE data-sharing requirements, as the data are currently stated to be available upon request rather than through a public repository.**  We would like to clarify that, due to privacy concerns regarding the farmers' dataset, we have made the data available upon request rather than through a public repository. Although the data is de-identified and anonymized, we are unable to share it publicly due to confidentiality and privacy considerations. PLOS ONE's data-sharing policy allows us to make their datasets available upon request, and we believe this approach complies with the journal's guidelines while protecting sensitive information. We are committed to providing access to the data in a manner that ensures the privacy of the respondents is maintained. |

Before AW_score was centered

After AW_score is centered
